# Supplementary material for: Racial and ethnic differentials in COVID-19-related job exposures by occupational standing in the US
Source: medRxiv. 2021 Apr 6:2020.11.13.20231431. Originally published 2020 Nov 16. Preprint. [Version 2] doi: 10.1101/2020.11.13.20231431 (PMC7685333; doi:10.1101/2020.11.13.20231431)
Supplement: Supplement 1 — S1 Appendix: Racial/ethnic distributions within each occupational subset displayed in main text figures [file media-1.pdf]

S1 Appendix: Racial/ethnic distributions within each occupational subset displayed in Main Text Figures

Updated April 5, 2021

Table S1.1: Racial/ethnic distribution of all recent workers by sex

Race/Ethnicity	Male		Female	
	N	Percent (%)	N	Percent (%)
White	675,542	63.1	632,421	62.4
Black	76,649	10.6	87,168	12.7
Latino	133,868	17.7	120,853	15.8
Chinese	13,095	1.3	14,265	1.5
Filipino	7,798	0.8	10,403	1.1
Vietnamese	5,150	0.6	5,519	0.6
Korean	4,303	0.5	4,684	0.5
Other Asian	22,072	2.5	18,654	2.2
Native American	7,018	0.6	7,059	0.6
Pacific Islander	1,402	0.2	1,353	0.2
Mixed Race	17,555	2.0	17,857	2.1
Other Race	1,695	0.2	1,778	0.2
Total	966,147	100	922,014	100

Note: Data are from the 2018 American Community Survey (ACS). Percentages are calculated using weights provided by the ACS; sample sizes (N) are unweighted. The percentages in this table correspond to those displayed in panel 1 in Figs 1-2.

Table S1.2: Racial/ethnic distribution of male frontline workers by quartile of occupational standing

Race/Ethnicity	Quartile of occupational standing					
	1st Quartile	2nd Quartile	3rd Quartile	4th Quartile	All frontline workers	Total N
White	53.6	62.1	71.2	69.6	59.0	399,608
Black	13.5	12.4	8.4	6.6	12.2	55,605
Latino	26.6	17.8	12.9	8.5	21.6	101,260
Chinese	0.7	0.9	1.0	2.5	0.9	5,257
Filipino	0.6	0.9	0.8	2.8	0.8	4,751
Vietnamese	0.5	0.8	0.4	1.0	0.6	3,236
Korean	0.2	0.4	0.5	1.1	0.3	1,881
Other Asian	1.3	1.7	2.1	5.3	1.7	9,240
Native American	0.8	0.6	0.5	0.3	0.7	5,329
Pacific Islander	0.2	0.2	0.1	0.1	0.2	1,009
Mixed Race	1.9	2.0	2.0	2.1	1.9	11,022
Other Race	0.2	0.2	0.2	0.3	0.2	1,077
Total Percent	100	100	100	100	100	
Total N	327,280	154,040	91,625	26,330	599,275	

Note: Data are from the 2018 American Community Survey (ACS). Percentages are calculated using weights provided by the ACS; sample sizes (N) are unweighted. Occupational standing is defined as the percentage of ACS respondents reporting this occupation title who completed at least one year of college education. Occupational standing is then divided into quartiles. The 1st quartile represents the lowest quartile of occupational standing and the 4th quartile the highest quartile of occupational standing. The percentages in this table correspond to those displayed in panel 2 of Figs 1 and 3.

Table S1.3: Racial/ethnic distribution of female frontline workers by quartile of occupational standing

Race/Ethnicity	Quartile of occupational standing					
	1st Quartile	2nd Quartile	3rd Quartile	4th Quartile	All frontline workers	Total N
White	47.6	55.2	65.8	71.7	57.0	285,517
Black	15.7	17.0	11.8	9.4	14.5	48,456
Latino	28.3	18.1	14.3	7.2	19.3	70,995
Chinese	1.1	1.3	1.2	1.5	1.2	5,765
Filipino	1.0	1.3	1.1	3.0	1.4	6,232
Vietnamese	0.7	1.3	0.4	0.7	0.8	3,542
Korean	0.4	0.5	0.5	0.8	0.5	2,189
Other Asian	1.9	1.8	1.6	3.3	2.0	8,247
Native American	0.9	0.6	0.6	0.3	0.7	3,989
Pacific Islander	0.2	0.2	0.2	0.1	0.2	719
Mixed Race	2.1	2.3	2.2	1.9	2.2	8,948
Other Race	0.3	0.3	0.2	0.2	0.3	963
Total Percent	100	100	100	100	100	
Total N	146,643	140,283	92,265	66,371	445,562	

Note: Data are from the 2018 American Community Survey (ACS). Percentages are calculated using weights provided by the ACS; sample sizes (N) are unweighted. Occupational standing is defined as the percentage of ACS respondents reporting this occupation title who completed at least one year of college education. Occupational standing is then divided into quartiles. The 1st quartile represents the lowest quartile of occupational standing and the 4th quartile the highest quartile of occupational standing. The percentages in this table correspond to those displayed in panel 2 of Figs 2 and 4.

Table S1.4: Racial/ethnic distribution of male frontline workers at highest risk of exposure to infection by quartile of occupational standing

Race/Ethnicity	Quartile of occupational standing					
	1st Quartile	2nd Quartile	3rd Quartile	4th Quartile	All in risk quartile	Total N
White	51.7	57.4	65.4	67.2	59.1	71,144
Black	17.1	17.7	12.5	7.3	14.4	11,717
Latino	24.8	16.9	14.4	8.5	17.4	15,040
Chinese	0.6	0.9	0.8	2.7	1.1	1,234
Filipino	0.9	1.6	1.4	3.3	1.6	1,728
Vietnamese	0.4	0.7	0.4	1.1	0.6	628
Korean	0.3	0.4	0.2	1.1	0.5	487
Other Asian	1.0	1.4	1.5	6.0	2.2	2,187
Native American	1.1	0.7	0.7	0.3	0.7	1,120
Pacific Islander	0.2	0.2	0.2	0.1	0.2	190
Mixed Race	1.9	1.9	2.4	2.0	2.0	2,060
Other Race	0.2	0.3	0.3	0.3	0.2	200
Total Percent	100	100	100	100	100	
Total N	34,776	27,899	23,147	21,913	107,735	

Note: Data are from the 2018 American Community Survey (ACS). Percentages are calculated using weights provided by the ACS; sample sizes (N) are unweighted. Workers are considered to be at highest risk of exposure to infection if the average exposure to infection for their occupation in the O*NET data falls in the highest quartile of the occupations in the analysis. Occupational standing is defined as the percentage of ACS respondents reporting this occupation title who completed at least one year of college education. Occupational standing is then divided into quartiles. The 1st quartile represents the lowest quartile of occupational standing and the 4th quartile the highest quartile of occupational standing. The percentages in this table correspond to those displayed in panel 3 of Figs 1 and 3.

Table S1.5: Racial/ethnic distribution of male frontline workers at highest risk of physical proximity to others by quartile of occupational standing

Race/Ethnicity	Quartile of occupational standing					
	1st Quartile	2nd Quartile	3rd Quartile	4th Quartile	All in risk quartile	Total N
White	53.5	55.9	64.1	69.5	58.7	77,439
Black	9.0	14.9	12.3	6.5	11.6	10,374
Latino	31.9	19.1	14.5	8.4	20.0	18,645
Chinese	0.6	1.4	1.0	2.4	1.3	1,597
Filipino	0.6	1.3	1.7	3.1	1.5	1,711
Vietnamese	0.3	1.4	0.5	0.8	0.9	948
Korean	0.2	0.5	0.3	1.0	0.5	547
Other Asian	0.8	2.4	1.8	5.7	2.5	2,738
Native American	0.8	0.6	0.6	0.3	0.6	988
Pacific Islander	0.2	0.2	0.2	0.1	0.2	181
Mixed Race	1.9	2.1	2.7	2.1	2.1	2,427
Other Race	0.2	0.3	0.3	0.3	0.3	242
Total Percent	100	100	100	100	100	
Total N	28,853	50,772	17,301	20,911	11,7837	

Note: Data are from the 2018 American Community Survey (ACS). Percentages are calculated using weights provided by the ACS; sample sizes (N) are unweighted. Workers are considered to be at highest risk of physical proximity to others if the average level of physical proximity to others for their occupation in the O*NET data falls in the highest quartile of the occupations in the analysis. Occupational standing is defined as the percentage of ACS respondents reporting this occupation title who completed at least one year of college education. Occupational standing is then divided into quartiles. The 1st quartile represents the lowest quartile of occupational standing and the 4th quartile the highest quartile of occupational standing. The percentages in this table correspond to those displayed in panel 4 of Figs 1 and 3.

Table S1.6: Racial/ethnic distribution of male frontline workers at highest risk of face-to-face discussions by quartile of occupational standing

Race/Ethnicity	Quartile of occupational standing					
	1st Quartile	2nd Quartile	3rd Quartile	4th Quartile	All in risk quartile	Total N
White	56.0	67.4	70.6	68.3	64.1	111,245
Black	14.8	9.3	8.2	6.6	10.7	12,335
Latino	23.4	17.2	13.0	7.6	17.4	21,536
Chinese	0.4	0.7	1.0	2.9	0.9	1,460
Filipino	0.6	0.6	0.8	3.3	0.9	1,432
Vietnamese	0.3	0.4	0.4	1.2	0.5	689
Korean	0.2	0.3	0.7	1.1	0.4	659
Other Asian	1.0	1.3	2.8	6.5	2.1	2,996
Native American	0.7	0.6	0.5	0.2	0.6	1,104
Pacific Islander	0.2	0.1	0.1	0.1	0.2	238
Mixed Race	2.2	1.8	1.7	2.0	2.0	2,826
Other Race	0.2	0.2	0.2	0.3	0.2	265
Total Percent	100	100	100	100	100	
Total N	54,074	48,152	36,232	18,327	156,785	

Note: Data are from the 2018 American Community Survey (ACS). Percentages are calculated using weights provided by the ACS; sample sizes (N) are unweighted. Workers are considered to be at highest risk of face-to-face discussions if the average level of face-to-face discussions for their occupation in the O*NET data falls in the highest quartile of the occupations in the analysis. Occupational standing is defined as the percentage of ACS respondents reporting this occupation title who completed at least one year of college education. Occupational standing is then divided into quartiles. The 1st quartile represents the lowest quartile of occupational standing and the 4th quartile the highest quartile of occupational standing. The percentages in this table correspond to those displayed in panel 5 of Figs 1 and 3.

Table S1.7: Racial/ethnic distribution of male frontline workers at highest risk of interactions with external customers or the public by quartile of occupational standing

Race/Ethnicity	Quartile of occupational standing					
	1st Quartile	2nd Quartile	3rd Quartile	4th Quartile	All in risk quartile	Total N
White	51.8	63.1	73.2	71.2	67.5	96,530
Black	14.4	11.5	7.2	5.6	9.3	8,933
Latino	21.6	17.7	12.6	8.0	15.0	16,183
Chinese	1.2	0.9	0.9	3.0	1.0	1,449
Filipino	1.0	0.9	0.6	2.1	0.8	985
Vietnamese	0.7	0.5	0.3	1.7	0.5	613
Korean	0.6	0.4	0.5	1.3	0.5	660
Other Asian	4.3	1.6	2.0	4.8	2.3	2,710
Native American	0.8	0.6	0.5	0.1	0.5	825
Pacific Islander	0.1	0.1	0.1	0.1	0.1	166
Mixed Race	3.3	2.4	1.9	1.8	2.2	2,700
Other Race	0.2	0.3	0.2	0.2	0.2	227
Total Percent	100	100	100	100	100	
Total N	14,600	39,186	71,092	7,103	131,981	

Note: Data are from the 2018 American Community Survey (ACS). Percentages are calculated using weights provided by the ACS; sample sizes (N) are unweighted. Workers are considered to be at highest risk of interactions with external customers or the public if the average level of interactions with external customers or the public for their occupation in the O*NET data falls in the highest quartile of the occupations in the analysis. Occupational standing is defined as the percentage of ACS respondents reporting this occupation title who completed at least one year of college education. Occupational standing is then divided into quartiles. The 1st quartile represents the lowest quartile of occupational standing and the 4th quartile the highest quartile of occupational standing. The percentages in this table correspond to those displayed in panel 6 of Figs 1 and 3.

Table S1.8: Racial/ethnic distribution of male frontline workers at highest risk of working indoors by quartile of occupational standing

Race/Ethnicity	Quartile of occupational standing					
	1st Quartile	2nd Quartile	3rd Quartile	4th Quartile	All in risk quartile	Total N
White	50.8	52.6	58.2	67.4	62.2	22,561
Black	15.8	11.2	14.1	7.1	9.8	2,371
Latino	27.2	21.8	16.2	8.5	13.7	3,638
Chinese	0.9	2.7	1.4	3.0	2.4	813
Filipino	0.5	1.5	3.2	3.0	2.5	796
Vietnamese	1.1	2.0	0.6	1.2	1.2	373
Korean	0.4	1.2	0.3	1.2	1.0	328
Other Asian	1.1	3.3	2.1	6.1	4.5	1,409
Native American	0.7	1.0	0.6	0.3	0.5	185
Pacific Islander	0.1	0.5	0.2	0.1	0.2	44
Mixed Race	1.3	2.0	2.7	1.9	2.0	628
Other Race	0.2	0.3	0.4	0.3	0.3	67
Total Percent	100	100	100	100	100	
Total N	4,281	3,596	4,332	21,004	33,213	

Note: Data are from the 2018 American Community Survey (ACS). Percentages are calculated using weights provided by the ACS; sample sizes (N) are unweighted. Because working indoors rather than outdoors reflects high risk, we take a maximum of the O*NET values for two variables measuring working outdoors and designate workers whose occupations fall in the lowest quartile of this measure as having the highest risk of working indoors. Occupational standing is defined as the percentage of ACS respondents reporting this occupation title who completed at least one year of college education. Occupational standing is then divided into quartiles. The 1st quartile represents the lowest quartile of occupational standing and the 4th quartile the highest quartile of occupational standing. The percentages in this table correspond to those displayed in panel 7 of Figs 1 and 3.

Table S1.9: Racial/ethnic distribution of female frontline workers at highest risk of exposure to infection by quartile of occupational standing

Race/Ethnicity	Quartile of occupational standing					
	1st Quartile	2nd Quartile	3rd Quartile	4th Quartile	All in risk quartile	Total N
White	38.0	52.7	59.2	71.8	57.3	129,213
Black	15.3	21.3	16.2	9.5	15.8	23,792
Latino	40.2	18.0	16.4	7.1	18.0	29,777
Chinese	0.8	1.1	1.0	1.5	1.1	2,379
Filipino	1.1	1.4	1.6	3.0	1.9	3,819
Vietnamese	0.4	0.5	0.4	0.7	0.5	1,053
Korean	0.2	0.3	0.4	0.8	0.5	959
Other Asian	1.1	1.5	1.6	3.2	2.0	3,703
Native American	1.0	0.7	0.7	0.3	0.6	1,726
Pacific Islander	0.2	0.2	0.1	0.1	0.2	290
Mixed Race	1.3	2.0	2.1	1.8	1.9	3,514
Other Race	0.5	0.3	0.3	0.2	0.3	459
Total Percent	100	100	100	100	100	
Total N	29,869	68,645	36,755	65,415	200,684	

Note: Data are from the 2018 American Community Survey (ACS). Percentages are calculated using weights provided by the ACS; sample sizes (N) are unweighted. Workers are considered to be at highest risk of exposure to infection if the average exposure to infection for their occupation in the O*NET data falls in the highest quartile of the occupations in the analysis. Occupational standing is defined as the percentage of ACS respondents reporting this occupation title who completed at least one year of college education. Occupational standing is then divided into quartiles. The 1st quartile represents the lowest quartile of occupational standing and the 4th quartile the highest quartile of occupational standing. The percentages in this table correspond to those displayed in panel 3 of Figs 2 and 4.

Table S1.10: Racial/ethnic distribution of female frontline workers at highest risk of physical proximity to others by quartile of occupational standing

Race/Ethnicity	Quartile of occupational standing					
	1st Quartile	2nd Quartile	3rd Quartile	4th Quartile	All in risk quartile	Total N
White	56.2	53.2	62.0	72.6	60.2	137,670
Black	12.7	18.5	14.4	9.2	14.9	22,852
Latino	22.6	18.1	15.6	7.0	15.0	26,002
Chinese	1.2	1.5	0.9	1.4	1.3	2,786
Filipino	0.9	1.4	1.5	3.1	1.8	3,768
Vietnamese	0.6	1.7	0.4	0.5	1.1	2,094
Korean	0.4	0.5	0.4	0.7	0.5	1,041
Other Asian	1.5	1.8	1.5	3.0	2.0	3,973
Native American	0.6	0.7	0.7	0.3	0.6	1,663
Pacific Islander	0.3	0.2	0.1	0.1	0.2	289
Mixed Race	2.9	2.3	2.3	1.8	2.2	4,119
Other Race	0.2	0.3	0.2	0.2	0.3	438
Total Percent	100	100	100	100	100	
Total N	12,673	93,583	41,868	58,571	206,695	

Note: Data are from the 2018 American Community Survey (ACS). Percentages are calculated using weights provided by the ACS; sample sizes (N) are unweighted. Workers are considered to be at highest risk of physical proximity to others if the average level of physical proximity to others for their occupation in the O*NET data falls in the highest quartile of the occupations in the analysis. Occupational standing is defined as the percentage of ACS respondents reporting this occupation title who completed at least one year of college education. Occupational standing is then divided into quartiles. The 1st quartile represents the lowest quartile of occupational standing and the 4th quartile the highest quartile of occupational standing. The percentages in this table correspond to those displayed in panel 4 of Figs 2 and 4.

Table S1.11: Racial/ethnic distribution of female frontline workers at highest risk of face-to-face discussions by quartile of occupational standing

Race/Ethnicity	Quartile of occupational standing					
	1st Quartile	2nd Quartile	3rd Quartile	4th Quartile	All in risk quartile	Total N
White	53.8	63.2	64.2	71.3	65.4	102,688
Black	16.6	11.5	14.0	9.6	12.0	12,479
Latino	21.6	16.9	13.8	6.9	12.9	15,405
Chinese	0.7	0.9	1.0	1.5	1.2	1,728
Filipino	0.9	1.0	1.2	3.2	1.9	2,633
Vietnamese	0.5	0.8	0.3	0.7	0.6	818
Korean	0.3	0.5	0.6	0.9	0.7	903
Other Asian	1.9	1.9	1.7	3.4	2.4	3,116
Native American	0.9	0.5	0.7	0.3	0.5	819
Pacific Islander	0.2	0.2	0.2	0.1	0.1	176
Mixed Race	2.3	2.4	2.1	1.9	2.1	2,660
Other Race	0.3	0.3	0.2	0.2	0.2	263
Total Percent	100	100	100	100	100	
Total N	14,683	36,851	36,626	55,528	143,688	

Note: Data are from the 2018 American Community Survey (ACS). Percentages are calculated using weights provided by the ACS; sample sizes (N) are unweighted. Workers are considered to be at highest risk of face-to-face discussions if the average level of face-to-face discussions for their occupation in the O*NET data falls in the highest quartile of the occupations in the analysis. Occupational standing is defined as the percentage of ACS respondents reporting this occupation title who completed at least one year of college education. Occupational standing is then divided into quartiles. The 1st quartile represents the lowest quartile of occupational standing and the 4th quartile the highest quartile of occupational standing. The percentages in this table correspond to those displayed in panel 5 of Figs 2 and 4.

Table S1.12: Racial/ethnic distribution of female frontline workers at highest risk of interactions with external customers or the public by quartile of occupational standing

Race/Ethnicity	Quartile of occupational standing					
	1st Quartile	2nd Quartile	3rd Quartile	4th Quartile	All in risk quartile	Total N
White	51.9	60.9	68.4	72.7	62.1	91,397
Black	17.7	13.2	9.2	6.9	12.5	12,346
Latino	21.0	17.3	14.4	6.7	16.4	18,336
Chinese	1.3	1.0	1.2	2.1	1.2	1,793
Filipino	1.0	1.0	0.9	1.9	1.0	1,333
Vietnamese	0.5	0.8	0.4	1.5	0.6	798
Korean	0.5	0.5	0.6	1.5	0.6	811
Other Asian	2.1	1.9	1.7	4.4	2.1	2,607
Native American	1.0	0.6	0.6	0.3	0.7	1,093
Pacific Islander	0.2	0.2	0.2	0.1	0.2	216
Mixed Race	2.7	2.4	2.3	1.7	2.4	2,950
Other Race	0.2	0.3	0.2	0.2	0.2	261
Total Percent	100	100	100	100	100	
Total N	33,212	41,527	48,165	11,037	133,941	

Note: Data are from the 2018 American Community Survey (ACS). Percentages are calculated using weights provided by the ACS; sample sizes (N) are unweighted. Workers are considered to be at highest risk of interactions with external customers or the public if the average level of interactions with external customers or the public for their occupation in the O*NET data falls in the highest quartile of the occupations in the analysis. Occupational standing is defined as the percentage of ACS respondents reporting this occupation title who completed at least one year of college education. Occupational standing is then divided into quartiles. The 1st quartile represents the lowest quartile of occupational standing and the 4th quartile the highest quartile of occupational standing. The percentages in this table correspond to those displayed in panel 6 of Figs 2 and 4.

Table S1.13: Racial/ethnic distribution of female frontline workers at highest risk of working indoors by quartile of occupational standing

Race/Ethnicity	Quartile of occupational standing					
	1st Quartile	2nd Quartile	3rd Quartile	4th Quartile	All in risk quartile	Total N
White	51.6	62.9	61.7	71.0	67.0	67,563
Black	8.4	12.3	16.2	9.8	11.5	7,816
Latino	26.7	15.6	14.1	7.1	10.6	8,249
Chinese	3.2	1.3	0.8	1.5	1.4	1,327
Filipino	0.9	0.7	1.8	3.1	2.4	2,192
Vietnamese	2.4	1.6	0.3	0.8	0.8	724
Korean	1.8	0.8	0.3	0.9	0.8	691
Other Asian	3.0	2.1	1.4	3.4	2.7	2,321
Native American	0.3	0.5	0.8	0.3	0.5	520
Pacific Islander	0.1	0.1	0.1	0.1	0.1	81
Mixed Race	1.4	2.0	2.3	1.9	2.0	1,614
Other Race	0.1	0.2	0.3	0.2	0.2	156
Total Percent	100	100	100	100	100	
Total N	3,777	12,149	19,573	57,755	93,254	

Note: Data are from the 2018 American Community Survey (ACS). Percentages are calculated using weights provided by the ACS; sample sizes (N) are unweighted. Because working indoors rather than outdoors reflects high risk, we take a maximum of the O*NET values for two variables measuring working outdoors and designate workers whose occupations fall in the lowest quartile of this measure as having the highest risk of working indoors. Occupational standing is defined as the percentage of ACS respondents reporting this occupation title who completed at least one year of college education. Occupational standing is then divided into quartiles. The 1st quartile represents the lowest quartile of occupational standing and the 4th quartile the highest quartile of occupational standing. The percentages in this table correspond to those displayed in panel 7 of Figs 2 and 4.
