# Supplementary material for: Racial and ethnic differentials in COVID-19-related job exposures by occupational standing in the US
Source: medRxiv. 2021 Apr 6:2020.11.13.20231431. Originally published 2020 Nov 16. Preprint. [Version 2] doi: 10.1101/2020.11.13.20231431 (PMC7685333; doi:10.1101/2020.11.13.20231431)
Supplement: Supplement 2 — S2 Appendix: Common high-risk occupations by occupational standing quartile [file media-2.pdf]

S2 Appendix: Common high-risk occupations by occupational standing quartile

Updated April 5, 2021

Table S2.1: Common high-risk occupations held by males by occupational standing quartile

Occupational standing Quartile	Occupations at highest risk of exposure to infection	Percent
1st quartile	janitors and building cleaners	62.8
	pipelayers, plumbers, pipefitters, and steamfitters	18.4
	maids and housekeeping cleaners	6.7
2nd quartile	maintenance and repair workers, general	21.9
	bus and ambulance drivers and attendants	14.0
	personal care aides	13.7
	sheriffs, bailiffs, correctional officers, and jailers	11.9
	nursing, psychiatric and home health aides	10.7
	first-line supervisors of housekeeping and janitorial workers	5.9
	telecommunications line installers and repairers	5.4
3rd quartile	police officers and detectives	35.9
	firefighters	13.6
	medical assistants and other healthcare support occupations, other	7.3
	emergency medical technicians and paramedics	7.0
	health diagnosing and treating practitioner support technicians	6.7
	licensed practical and licensed vocational nurses	5.7
4th quartile	physicians and surgeons	30.6
	registered nurses	21.7
	pharmacists	7.2
	diagnostic related technologists and technicians	6.8
	dentists	6.0
	clinical laboratory technologists and technicians	5.1
	**Occupations at highest risk of physical proximity to others**	
1st quartile	carpenters	45.4
	combined food preparation and serving workers, including fast food	9.0
	food preparation and serving related workers, other	8.7
	roofers	8.5
	butchers and other meat, poultry, and fish processing workers	6.8
	brickmasons, blockmasons, and stonemasons	6.1
2nd quartile	first-line supervisors of construction trades and extraction workers	17.4
	waiters and waitresses	15.9
	taxi drivers and chauffeurs	13.1
	bus and ambulance drivers and attendants	7.5

	industrial and refractory machinery mechanics	7.4
	personal care aides	7.3
	sheriffs, bailiffs, correctional officers, and jailers	6.4
	nursing, psychiatric, and home health aides	5.7
	first-line supervisors of food preparation and serving workers	5.3
3rd quartile	firefighters	17.7
	recreation and fitness workers	14.2
	bartenders	11.9
	medical assistants and other healthcare support occupations, other	9.5
	emergency medical technicians and paramedics	9.1
	health diagnosing and treating practitioner support technicians	8.7
	licensed practical and licensed vocational nurses	7.5
	first-line supervisors of police and detectives	6.1
4th quartile	physicians and surgeons	32.4
	registered nurses	22.9
	aircraft pilots and flight engineers	9.8
	diagnostic related technologists and technicians	7.1
	dentists	6.3
	**Occupations at highest risk of face-to-face discussions**	
1st quartile	stock clerks and order fillers	24.8
	carpenters	24.7
2nd quartile	retail salespersons	42.7
	first-line supervisors of construction trades and extraction workers	19.1
	maintenance and repair workers, general	12.9
	first-line supervisors of mechanics, installers, and repairers	6.0
	supervisors of transportation and material moving workers	5.4
3rd quartile	first-line supervisors of sales workers	78.2
	insurance sales agents	8.8
4th quartile	physicians and surgeons	36.9
	registered nurses	26.1
	pharmacists	8.6
	dentists	7.2
	**Occupations at highest risk of interactions with external customers or the public**	
1st quartile	cashiers	79.9
	automotive and watercraft service attendants	6.6
	vehicle and mobile equipment mechanics, installers, and repairers, other	6.3
2nd quartile	retail salespersons	50.3
	sheriffs, bailiffs, correctional officers, and jailers	8.2

	first-line supervisors of mechanics, installers, and repairers	7.1
	first-line supervisors of food preparation and serving workers	6.9
	law enforcement workers, other	5.5
3rd quartile	first-line supervisors of sales workers	40.1
	sales representatives, wholesale and manufacturing	15.3
	police officers and detectives	11.9
	sales representatives, services, all other	7.4
4th quartile	pharmacists	22.6
	diagnostic related technologists and technicians	21.3
	dentists	18.9
	physical therapists	12.3
	respiratory therapists	6.5
	sales engineers	5.7
	veterinarians	5.6
	**Occupations at highest risk of working indoors**	
1st quartile	bookbinders, printing machine operators, and job printers	32.2
	barbers	24.8
	sewing machine operators	11.7
	upholsterers	6.3
	extruding, forming, pressing, and compacting machine setters, operators, and tenders	6.3
2nd quartile	bakers	26.4
	hairdressers, hairstylists, and cosmetologists	22.0
	gaming services workers	18.0
	medical, dental, and ophthalmic laboratory technicians	11.9
	telemarketers	9.0
	jewelers and precious stone and metal workers	6.6
3rd quartile	bartenders	44.8
	licensed practical and licensed vocational nurses	28.1
	health technologists and technicians, other	10.9
4th quartile	physicians and surgeons	32.0
	registered nurses	22.6
	securities, commodities, and financial services sales agents	9.4
	pharmacists	7.5
	diagnostic related technologists and technicians	7.1
	dentists	6.3
	clinical laboratory technologists and technicians	5.3

Note: Occupational standing is defined as the percentage of ACS respondents reporting this occupation title who completed at least one year of college education. Occupational standing is then divided into quartiles. The 1st quartile represents the lowest quartile of occupational standing and the 4th quartile the highest quartile of occupational standing. Information on the five occupational risk factors for COVID-19 is obtained from O*NET; occupations in the top (4th) quartile for each of these risk factors are considered to be at highest risk. Values are the

percentage of workers employed in the particular combination of occupational standing quartile and risk factor quartile who report the listed job title. For brevity, only occupations exceeding 5% are displayed.

Table S2.2: Common high-risk occupations held by females by occupational standing quartile

Occupational standing Quartile	Occupations at highest risk of exposure to infection	Percent
1st Quartile	maids and housekeeping cleaners	52.9
	janitors and building cleaners	35.8
	food servers, nonrestaurant	5.0
2nd Quartile	nursing, psychiatric, and home health aides	29.0
	childcare workers	23.1
	personal care aides	22.7
	hairdressers, hairstylists, and cosmetologists	12.4
3rd Quartile	medical assistants and other healthcare support occupations, other	26.0
	licensed practical and licensed vocational nurses	24.2
	health diagnosing and treating practitioner support technicians	17.8
	dental assistants	8.6
4th Quartile	registered nurses	56.7
	physicians and surgeons	6.2
	**Occupations at highest risk of physical proximity to others**	
1st Quartile	combined food preparation and serving workers, including fast food	37.2
	host and hostesses, restaurant, lounge, and coffee shop	29.0
	food preparation and serving related workers, other	13.9
	butchers and other meat, poultry, and fish processing workers	6.2
2nd Quartile	nursing, psychiatric, and home health aides	21.0
	waiters and waitresses	20.3
	childcare workers	16.7
	personal care aides	16.5
	hairdressers, hairstylists, and cosmetologists	9.0
3rd Quartile	medical assistants and other healthcare support occupations, other	22.9
	licensed practical and licensed vocational nurses	21.4
	health diagnosing and treating practitioner support technicians	15.7
	recreation and fitness workers	9.9
	dental assistants	7.6
	bartenders	7.5
4th Quartile	registered nurses	63.7
	physicians and surgeons	6.9
	diagnostic related technologists and technicians	5.5
	**Occupations at highest risk of face-to-face discussions**	
1st Quartile	stock clerks and order fillers	51.7
	laborers and freight, stock, and material movers, hand	42.4
2nd Quartile	retail salespersons	60.1

	hairdressers, hairstylists, and cosmetologists	23.7
	nonfarm animal caretakers	7.6
3rd Quartile	first-line supervisors of sales workers	59.4
	licensed practical and licensed vocational nurses	25.2
	insurance sales agents	8.2
4th Quartile	registered nurses	66.8
	physicians and surgeons	7.3
	**Occupations at highest risk of interactions with external customers or the public**	
1st Quartile	cashiers	97.9
2nd Quartile	retail salespersons	53.3
	hairdressers, hairstylists, and cosmetologists	21.1
	first-line supervisors of food preparation and serving workers	9.4
	law enforcement workers, other	5.3
3rd Quartile	first-line supervisors of sales workers	45.0
	sales representatives, wholesale and manufacturing	9.3
	dental assistants	6.8
	bartenders	6.6
	insurance sales agents	6.2
	door-to-door sales workers, news and street vendors, and related workers	6.2
	sales representatives, services, all other	5.1
4th Quartile	diagnostic related technologists and technicians	28.8
	pharmacists	19.6
	physical therapists	17.9
	dieticians and nutritionists	10.5
	respiratory therapists	7.7
	dentists	5.8
	veterinarians	5.6
	**Occupations at highest risk of working indoors**	
1st Quartile	sewing machine operators	43.6
	tailors, dressmakers, and sewers	19.1
	bookbinders, printing machine operators, and job printers	15.5
	barbers	8.6
2nd Quartile	hairdressers, hairstylists, and cosmetologists	71.0
	bakers	13.7
3rd Quartile	licensed practical and licensed vocational nurses	46.2
	dental assistants	16.4
	bartenders	16.1
	medical records and health information technicians	9.4
	health technologists and technicians, other	5.5
4th Quartile	registered nurses	64.3

physicians and surgeons	7.0
diagnostic related technologists and technicians	5.5
clinical laboratory technologists and technicians	5.2

Note: Occupational standing is defined as the percentage of ACS respondents reporting this occupation title who completed at least one year of college education. Occupational standing is then divided into quartiles. The 1st quartile represents the lowest quartile of occupational standing and the 4th quartile the highest quartile of occupational standing. Information on the five occupational risk factors for COVID-19 is obtained from O*NET; occupations in the top (4th) quartile for each of these risk factors are considered to be at highest risk. Values are the percentage of workers employed in the particular combination of occupational standing quartile and risk factor quartile who report the listed job title. For brevity, only occupations exceeding 5% are displayed.
